# Mathematical Modeling of Drain Current Estimation in a CSDG MOSFET, Based on La_2_O_3_ Oxide Layer with Fabrication—A Nanomaterial Approach

**DOI:** 10.3390/nano12193374

**Published:** 2022-09-27

**Authors:** Naveenbalaji Gowthaman, Viranjay M. Srivastava

**Affiliations:** Department of Electronic Engineering, Howard College, University of KwaZulu-Natal, Durban 4041, South Africa

**Keywords:** cylindrical structure, double-gate MOSFET, drain current, nanotechnology, material properties, microelectronics, semiconductor, CSDG MOSFET, VLSI

## Abstract

In this work, three-dimensional modeling of the surface potential along the cylindrical surrounding double-gate (CSDG) MOSFET is proposed. The derived surface potential is used to predict the values of electron mobility along the length of the device, thereby deriving the drain current equation at the end of the device. The expressions are used for modeling the symmetric doped and undoped channel CSDG MOSFET device. This model uses Pao-Sah’s double integral to derive the current equation for the concentric cylindrical structure of the CSDG MOSFET. The three-dimensional surface potential estimation is performed analytically for doped and undoped device parameters. The maximum oxidant concentration of the oxide layer is observed to be 4.37 × 10^16^ cm^−3^ of the thickness of 0.82 nm for (100) and 3.90 × 10^16^ cm^−3^ of the thickness of 0.96 nm for (111) for dry oxidation, and 2.56 × 10^19^ cm^−3^ of thickness 0.33 nm for (100) and 2.11 × 10^19^ cm^−3^ of thickness 0.49 nm for (111) for wet oxidation environment conditions. Being an extensive analytical approach, the drain current serves the purpose of electron concentration explicitly inside the concentric cylindrical structures. The behavior of the device is analyzed for various threshold conditions of the gate voltage and other parameters.

## 1. Introduction

In recent years, the double-gate (DG) MOSFET design has been prominent in planar design structures. However, the limitations of the double-gate MOSFET have put an end to further advances in the regime [1]. New device structures are needed to overcome the conventional structures’ issues and enhance performance. Some new devices concentrate more on solving performance issues oriented with the short channel effects (SCEs) [2,3]. Most of the new devices are silicon-on-insulator (SOI) based on the packaging density and the suppressing capacity of the SCEs. Many multiple gate structures have been proposed in recent times, such as double-gate, tri-gate FET, gate-all-around FET, cylindrical gate-all-around FET, and the CSDG MOSFET [4,5,6]. Device modeling is the fundamental step of designing a device since it has the capacity to better understand the characteristics of the FET under various theoretical and boundary conditions. The multi-gate FET has been a promising feature of new-age devices to enhance performance and improve immunity to SCEs. Significant works have been undertaken by various researchers in the field of the double-gate with symmetrical and asymmetrical geometry [7,8,9,10]. Among these, symmetric geometrical structures are modeled in this work and analyzed for the improvement of their characteristics.

In the existing DG FET models [11], approximations with numerical iterations are used to derive the results from the mathematical models. The mobility model has been developed for all operation regions of a transistor. In all modeling, the transistor with an undoped structure has been modeled, with doping concentration added to reduce the complexity in computation. Deyasi et al. [12] developed a two-dimensional structure of DGFET based on the Ortiz-Conde model to overcome the SCEs involved in the conventional FETs.

Gowthaman and Srivastava [6] proposed an analytical model for the capacitance present in the lightly doped cylindrical surrounding double-gate MOSFET. Capacitive modeling was performed for this cylindrical structure. This modeling was analyzed for all operating regions of the transistors [13], capacitance estimation, and electrical field dependence on the capacitance. The results were compiled in a report, and recordings were tabulated. Horii et al. [14] developed a single-input, dual-output (SIDO) with a digital gate driver (DGD) integrated circuit, which works in a six-digit driver circuit. Moreover, it proposes the drain current (I_D_) with variable silicon-based MOSFETs. The direct current characteristics were analyzed using the gate control with amplitudes of voltage and ON-current, respectively. Gowthaman [15] worked in a cylindrical surrounding double-gate MOSFET to reduce the short channel effects by inserting high-ƙ dielectric in the oxide layer. The conventional dielectric material of silicon dioxide was replaced with lanthanum or lanthanum dioxide (La_2_O_3_) as a high-ƙ dielectric material. The gate oxide thickness was a great concern in designing the cylindrical surrounding double-gate MOSFET with high immunity towards SCEs. The ON-state and OFF-state current show considerable improvement in the design with the use of a CSDG MOSFET with a high-ƙ dielectric material to improve the controllability and observability.

Robertson [16] designed a conventional MOSFET that uses scaling to reduce the gate thickness of 1.4 nm equivalent to EOT. This results in a larger leakage current, making the physical oxide layer with high-ƙ dielectric material highly suitable for a CSDG MOSFET. Gaidhane et al. [17] analyzed the problems of gate-induced drain leakage (GIDL) associated with the negative capacitance effect of FinFET in 3D technology using TCAD simulations. A comprehensive analysis of the electronic parameters was presented in 7 nm technology by varying the ferroelectric and silicon body thickness. Chang et.al. [18] performed an analysis on the nickel gallium arsenide (Ni-InGaAs) alloy with source and drain terminal with low RSD with uniform depletion. Furthermore, the temperature dependency of the junction-less transistor was analyzed.

In this work, the symmetric MOSFET with a doped and undoped channel is modeled extensively based on the surface potential along with the cylindrical structure, mobility of electrons inside the channel, and the drain current measured at the terminal. This paper is organized as follows, Section 2 elaborates on modeling with a fabrication perspective and is supported by the equations to all electrical parameters. Section 3 deals with the fabrication steps performed in the cylindrical surrounding double-gate MOSFET paradigm. The results obtained from the simulation and the mathematical analysis are compared and inferences made in Section 4. Finally, Section 5 concludes the paper and recommends future consideration of work with useful insights.

## 2. Extensive Modeling of the CSDG MOSFET for Fabrication Perspective

**Figure 1:**
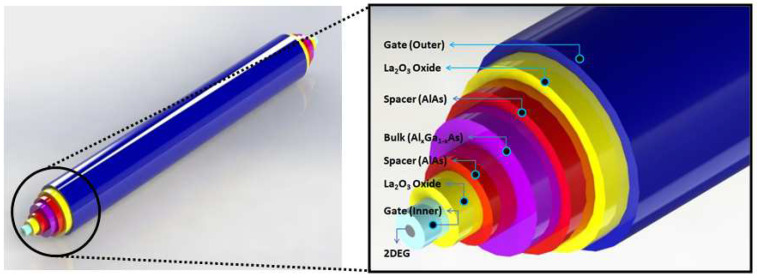
The CSDG MOSFET device showing the cylindrical layer structure [6].

The CSDG MOSFET has evolved from the basic double-gate MOSFET by taking the rotational axis outside the device, as shown in Figure 1. This resultant three-dimensional device will be coordinated in cylindrical dimensions [19,20]. The device follows the concentric cylindrical geometry with symmetry along the length of the device [21,22,23]. The outer gate layer is represented by the blue color. The next layer adjacent to the gate is a yellow oxide layer, followed by a spacer in red. The bulk is represented as a pink layer where the dopant concentration is optimum. The next layer to the bulk is another small layer of spacer and oxide that leads to the second gate at the center. The inner gate is in pale blue. The inner gate has a small core of 2D electron gas (2DEG) which reduces the skin effect and maintains electric potential to flow in the inner gate. The 2DEG layer provides enough flow path to the charge carriers.

The proposed structure modeling involves the following assumptions: Silicon body floats; hence, the electrons possess energies in Fermi energy levels, and the Fermi energy level of the source terminal is considered for all mathematical modeling [24]. The threshold voltage of the proposed device is derived analytically to simplify the drain current expression [25,26,27,28,29]. Firstly, the FET switches the layers on with lower threshold voltages and excites the electrons to the Fermi energy level [28]. Secondly, the layers with higher threshold voltage are turned on, and the energy excitation is modeled. The position along the length of the channel where the potential is low is called a virtual cathode. It is present in the channel to create mobility of electrons. The surface potential is calculated from this point that extends inside the core of the FET [30,31,32,33,34].

**Figure 2:**
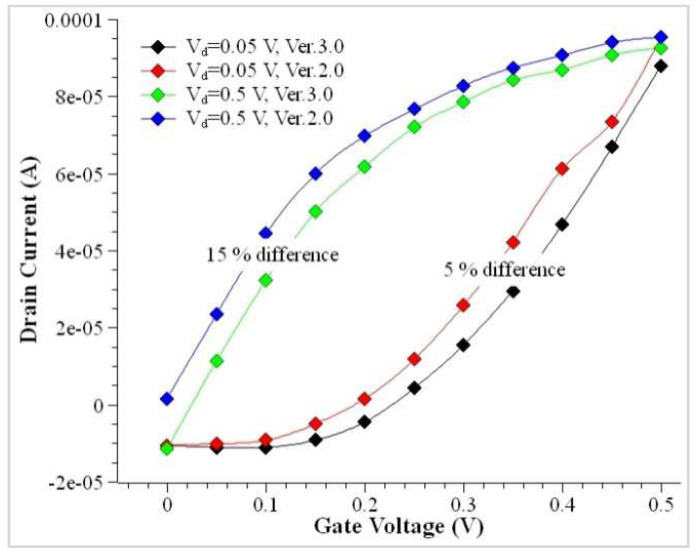
Comparison of the current environment with the conventional tool.

The modeling was performed analytically, and the simulation was coordinated and performed using an electronic simulator. The previous version of the simulation tool utilized higher system capacity and had limitations to developing the characteristics. However, the newer version has the potential to simulate the device with added features to simulate various characteristics of the device with a considerable amount of performance enhancement. Figure 2 shows the enhancement in the performance of the two different versions of the simulation tool. In lower current characteristics, the newer version shows a bigger difference than the older version. 

The proposed structure of the CSDG MOSFET is a cylindrical concentric solid device with various layers which extends on all the three axes. The capacitive modeling and its estimation were carried out by Gowthaman et al. in [6]. The three-dimensional surface potential of the cylindrical structure, drain current flows in the CSDG and the electron mobility model are discussed analytically. Table A1 shows the notations of the symbols used in this work.

### 2.1. Three-Dimensional Surface Potential Modeling

The 3D Poisson’s distribution for the three-dimensional CSDG MOSFET is given as:

(1) $$\frac{\partial^{2}\varphi \left(x,y,z\right)}{\partial x^{2}}+\frac{\partial^{2}\varphi \left(x,y,z\right)}{\partial y^{2}}+\frac{\partial^{2}\varphi \left(x,y,z\right)}{\partial z^{2}}=\frac{q}{\varepsilon s_{i}}\eta_{i}\exp\left[\frac{\psi \left(x\right)-V\left(y\right)+\psi \left(z\right)}{V_{t}}\right]$$

where the channel potential, *φ(x,y,z)* is given by

(2) $$\varphi \left(x,y,z\right)=\psi \left(x\right)+V\left(y\right)+\psi \left(z\right)$$

The potential used for band bending *ψ*(*x*) varies in the x-direction and the electrostatic potential *V*(*y*) fluctuates along the y-direction. Since the proposed structure extends in the *z*-direction, which is a circular disc, the net potential in the *z*-direction constitutes zero and is negligible. Moreover, *ψ*(*z*) = 0, *V*(0) = 0, and *V*(Leff) = *V_DS_*. The updated electrostatic potential across the y-direction along the channel length *L* is given as:

(3) $$V\left(y\right)=\frac{V_{DS}}{L}y$$

Utilizing (1) and (3) in (2), the equation of the channel potential subjected to the differential is given as:

(4) $$\frac{\partial^{2}\psi \left(x\right)}{\partial x^{2}}=\frac{q}{\varepsilon s_{i}}\eta_{i}\exp\left[\frac{1}{V_{t}}\left\{\varphi (x)-\left(\frac{V_{DS}}{L}y\right)\right\}\right]$$

The electrostatic potential along the y-direction is reduced to *V*, and (4) can be given as:

(5) $$\frac{d^{2}\psi \left(x\right)}{dx^{2}}=\frac{q}{\varepsilon s_{i}}\eta_{i}\exp\left[\frac{1}{V_{t}}\left\{\varphi \left(x\right)-V\right\}\right]$$

The thin concentric layers of the CSDG MOSFET have an approximation for the bending potential, *ψ = ψ*0 and *dψ/dx* = 0. Then, (5) can be rewritten as:

(6a) $${\left(\frac{d\psi }{dx}\right)}^{2}=\frac{2KT}{\varepsilon s_{i}}\eta_{i}e^{\left(-\frac{qV}{kT}\right)}\left\{e^{\left(\frac{q\psi }{kT}\right)}-e^{\left(\frac{q\psi_{0}}{kT}\right)}\right\}$$

or

(6b) $$F=\frac{d\psi }{dx}=\sqrt{\frac{2KT}{\varepsilon s_{i}}\eta_{i}e^{\left(-\frac{qV}{kT}\right)}\left\{e^{\left(\frac{q\psi }{kT}\right)}-e^{\left(\frac{q\psi_{0}}{kT}\right)}\right\}}$$

After integrating (5) twice with applying boundary conditions, the expression of the band bending potential can be given as:

(7) $$\psi \left(x\right)=\psi_{0}-\frac{2KT}{q}\log\left[\cos\left(\sqrt{\frac{q^{2}\eta_{i}}{2\varepsilon s_{i}kT}e^{\left(\frac{q\left(\psi_{0}-V\right)}{kT}\right)}x}\right)\right]$$

By way of

$$\sqrt{\frac{q^{2}\eta_{i}}{2\varepsilon s_{i}kT}e^{\left(\frac{q\left(\psi_{0}-V\right)}{kT}\right)}x}=\frac{\pi }{180},\begin{array}{cc} for & x=\frac{t_{si}}{2} \end{array}$$

can result as:

(8) $$\psi_{0}=V+\frac{kT}{q}\ln\left(\frac{8\pi^{2}\varepsilon s_{i}kT}{32,400q^{2}t_{si}^{2}\eta_{i}}\right)$$

In the undoped CSDG MOSFET device, the mathematically derived surface potential by the independent arbitrary potential technique is given as:

(9) $$\psi_{s}=V^{\prime }+\frac{kT}{q}\ln\left(\frac{2C_{0}\left[V_{GS}-V_{T}\right]}{qt_{si}\eta_{i}}\right)$$

where $V_{arbitrary}=0$ at the source terminal, and $V_{arbitrary}=V_{DS}-V_{bi}$ is the drain terminal. The junction between the drain and the channel influences the depleted charge carriers, which is the reason for the presence of potential at the drain terminal. The threshold voltage, *V_T_*, is given by,

(10) $$V_{T}=V_{FB}+\phi_{fb}+V\left(y\right)$$

For a doped channel, the CSDG MOSFET behaves as per the 3D Poisson’s expression given in (1), but it has an additional term for the dopant concentration. The dopant concentration is directly proportional to the surface potential. Poisson’s expression for the doped device is given as:

(11) $$\frac{\partial^{2}\varphi \left(x,y,z\right)}{\partial x^{2}}+\frac{\partial^{2}\varphi \left(x,y,z\right)}{\partial y^{2}}+\frac{\partial^{2}\varphi \left(x,y,z\right)}{\partial z^{2}}=\frac{q}{\varepsilon s_{i}}\left[N_{a}+\eta_{i}\exp\left(\frac{\psi \left(x\right)-V}{V_{t}}\right)\right]$$

The surface potential is derived from (8) and is substituted in (11), giving:

(12) $$\frac{d^{2}\psi \left(x\right)}{dx^{2}}=\frac{q}{\varepsilon_{si}}\left\{N_{a}+\left[\eta_{i}\cdot \exp\left(\frac{\psi_{0}-V}{V_{t}}\right)\cdot \sec^{2}\left(\sqrt{\frac{q^{2}\eta_{i}}{2\varepsilon_{si}}e^{\frac{q}{kT}\left(\psi_{0}-V\right)}x}\right)\right]\right\}$$

Integrating (12) two times (double integration) with optimum boundary conditions suitable for the CSDG MOSFET, the band bending potential can be rewritten as:

(13a) $$\psi \left(x\right)=\frac{q}{2\varepsilon_{si}}N_{a}x^{2}+\frac{2kT}{q}\ln\left|\sec\left(\sqrt{\frac{q^{2}\eta_{i}}{2\varepsilon_{si}}e^{\frac{q}{kT}\left(\psi_{0}-V\right)}x}\right)\right|+\psi_{0}$$

The surface potential is derived from (13) by substituting the boundary condition as,

(13b) $$\begin{array}{cc} x=\frac{t_{si}}{2} & \begin{array}{cc} \Rightarrow & \psi \left(x\right)=\frac{q}{2\varepsilon_{si}}N_{a}{\left(\frac{t_{si}}{2}\right)}^{2}+\frac{2kT}{q}\ln\left|\sec\left(\sqrt{\frac{q^{2}\eta_{i}}{2\varepsilon_{si}}e^{\frac{q}{kT}\left(\psi_{0}-V\right)}\frac{t_{si}}{2}}\right)\right|+\psi_{0} \end{array} \end{array}$$

The surface potential of the CSDG MOSFET with a doped channel has been derived in (13b), and it is a function of dopant concentration Na and potential at the center of the core of the CSDG MOSFET *ψ*0. 

### 2.2. Mobility Modeling in the Cylindrical Structure

The effective electron mobility model for an undoped CSDG MOSFET which extends in 3D space is given as,

(14) $$\mu_{eff}=\frac{\mu_{si}^{e^{-}}}{1+\left(\frac{E_{vertical}}{E_{horizontal}}\right)}$$

This is the basic equation for deriving electron mobility in the CSDG MOSFET paradigm. Initially, the mobility model for the devices that have > 10 nm channel length has been discussed, and by adding suitable boundary conditions, two inferences have been made. The mobility of electrons in the silicon layer $\mu_{si}^{e^{-}}$ is 1500 cm^2^ V^−1^s^−1^. The effective mobility of electrons (*µ_eff_*) is given as:

(15a) $$\mu_{eff}=\frac{\mu_{si}^{e^{-}}}{1+\left[V_{DS}+V_{bi}+\left(\frac{-\psi_{S_{0}}}{\psi_{SL}-\psi_{S_{0}}}\right)\right]}$$

The electron mobility by considering the device operates just above the flat band voltage is given as:

(15b) $$\mu_{eff}=\frac{\mu_{si}^{e^{-}}}{\sqrt{1+\left(\frac{V_{DS}+V_{bi}-\psi_{S_{0}}}{\psi_{SL}-\psi_{S_{0}}}\right)}}$$

If the device operates in the sub-threshold region, (15b) becomes:

(16a) $$\mu_{eff}=\frac{\mu_{si}^{e^{-}}}{1+\left[\frac{2L\left(\psi_{S_{0}}-\psi_{00}\right)}{t_{si}\left(V_{bi}-\psi_{S_{0}}\right)}\right]}$$

For weak inversion region:

(16b) $$\mu_{eff}=\frac{\mu_{si}^{e^{-}}}{\sqrt{1+\left(\frac{V_{DS}+V_{bi}+\psi_{SL}-\psi_{S_{0}}}{\psi_{SL}-\psi_{S_{0}}}\right)}}$$

For moderate inversion regions:

(16c) $$\mu_{eff}=\frac{\mu_{si}^{e^{-}}}{\sqrt{1+\left[\frac{2L\left(\psi_{S_{0}}-\psi_{00}\right)}{t_{si}\left(V_{bi}-\psi_{S_{0}}\right)}\right]}}$$

For channel thickness <= 10 nm, the flat band voltage is less than the *V_GS_*, and it is displayed as:

(17) $$\mu_{eff}=\frac{\mu_{si}^{e^{-}}}{1+\left[V_{DS}+V_{bi}+\left(\frac{-\psi_{S_{0}}}{\psi_{SL}-\psi_{S_{0}}}\right)\right]}$$

*V_GS_* <= flat band voltage, (17) becomes:

(18) $$\mu_{eff}=\frac{\mu_{si}^{e^{-}}}{1+\left[\frac{2L\left(\psi_{S_{0}}-\psi_{00}\right)}{t_{si}\left(V_{bi}-\psi_{S_{0}}\right)}\right]}$$

These are the mobility variations present in the CSDG MOSFET with various conditions of voltage across gate and source terminals. The effective mobility is shown in (18) and is used for further derivation of the drain current.

### 2.3. Drain Current Modeling for the Cylindrical Structure

The drain current estimation follows Pao-Sah’s distribution theory which involves both drift and diffusion charge carriers along the length of the device [35,36,37,38]. The independent mobility of an electron has been derived from:

(19a) $$I_{D}=\mu \frac{W}{L}\int_{0}^{V_{DS}}Q\cdot dV$$

The mobility modeling is used in estimating the drain current in the cylindrical structure of the CSDG MOSFET. The effective mobility in (18) is applied to the three-dimensional space with independent position nature to obtain:

(19b) $$\begin{array}{ll} I_{DS}=0 & for\;\left(V_{GS}<V_{T}\right)\\ I_{D(linear)}=\frac{\mu_{n}}{2}C_{ox}\frac{W}{L}\left[2\left(V_{GS}-V_{T}\right)V_{DS}-V_{DS}^{2}\right] & for\;\left\{\left(V_{GS}\geq V_{T}\right)\left[V_{DS}-\left(V_{GS}-V_{T}\right)\right]\right\}\\ I_{D(saturation)}=\frac{\mu_{n}}{2}C_{ox}\frac{W}{L}\left[{\left(V_{GS}-V_{T}\right)}^{2}\left(1+\lambda V_{DS}\right)\right] & for\;\left(V_{GS}\geq V_{T}\right) \end{array}$$

This (19b) gives the elementary current equation of the transistor in several working regions [39,40]. The capacitance estimation was carried out by the authors in [6,8,40], and applied to (19b), it gives:

(20) $$C_{ox\_cyl}=\frac{2\pi k\varepsilon_{0}}{d}\left[\left(r_{c1}^{2}-r_{c2}^{2}\right)+h\left(r_{c1}-r_{c2}\right)\right]$$

However, (20) in (19) yields the current equation for the linear region of the transistor as:

(21) $$I=\frac{\pi \varepsilon_{0}k\mu_{n}W}{d\cdot L}\left\{\left[2\left(V_{GS}-V_{T}\right)V_{DS}-V_{DS}^{2}\right]\left[\left(r_{c1}^{2}-r_{c2}^{2}\right)+h\left(r_{c1}-r_{c2}\right)\right]\right\}$$

The fixed charge distribution is not present in the silicon-based devices [41]; hence, it is given as:

(22) $$Q=2\varepsilon_{s}H_{s}=-2C_{ox\_cyl}(V_{GF}-\psi_{s})$$

where *H_S_* is the surface electrical field [42], and two indicates the device has symmetrical geometry. Substituting (22) in (19a) gives:

(23) $$I_{D}=2\mu \frac{W}{L}\int_{0}^{V_{DS}}\int_{\psi_{00}}^{\psi_{s}}\frac{\left[2\varepsilon_{0}\left(V_{GF}-\psi_{s}\right)\right]n}{F}\;d\psi \cdot dV$$

where *η* is the intrinsic carrier concentration, *H* is the electric field at the drain terminal, and *κ* is the coupling coefficient [43]. These terms are given as:

(24) $$\begin{array}{l} \eta =\eta_{i}e^{\beta (\psi -V)}\\ H=-\sqrt{\left(\frac{2kT\eta_{i}}{\varepsilon_{s}}\right)e^{\beta (\psi -V)}+\kappa }\\ \kappa =-\left(\frac{2kT\eta_{i}}{\varepsilon_{s}}\right)e^{\beta (\psi_{0}-V)} \end{array}$$

The final equation of current at the drain terminal is:

(25) $$I_{D}=\mu \frac{W}{L}\left\{2C_{ox\_cyl}\left[V_{GF}\left(\psi_{SL}-\psi_{S_{0}}\right)-\frac{1}{2}\left(\psi_{SL}^{2}-\psi_{S_{0}}^{2}\right)\right]+4\frac{kT}{q}C_{ox\_cyl}\left(\psi_{SL}-\psi_{S_{0}}\right)+t_{si}kT\eta_{i}\left[e^{\beta \left(\psi_{0L}-V_{DS}\right)}-e^{\beta \psi_{00}}\right]\right\}$$

The equation for the final drain current can be substituted with (20) to be influenced by the oxide layer capacitance that exists in a cylindrical structure. 

## 3. Fabrication Model of the CSDG MOSFET

By considering the surface potential, mobility concentration, and drain current parameters, the novel CSDG MOSFET has been proposed. The fabrication steps follow atomic vapor deposition under various ion concentrations in the controlled chamber [8,44]. The CSDG MOSFET has been designed using lanthanum oxide as a gate oxide layer. This layer gives better immunity to the short channel effects (SCEs).

**Figure 3:**
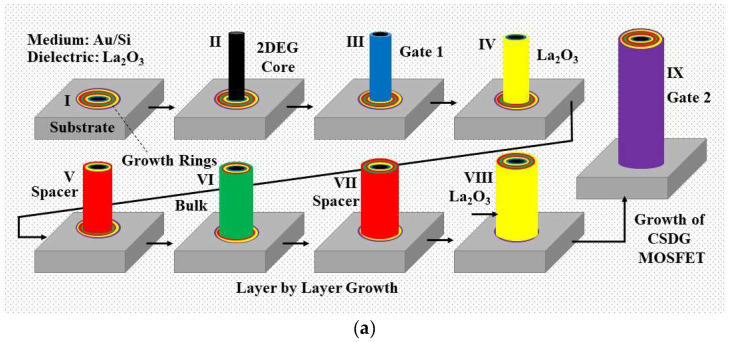

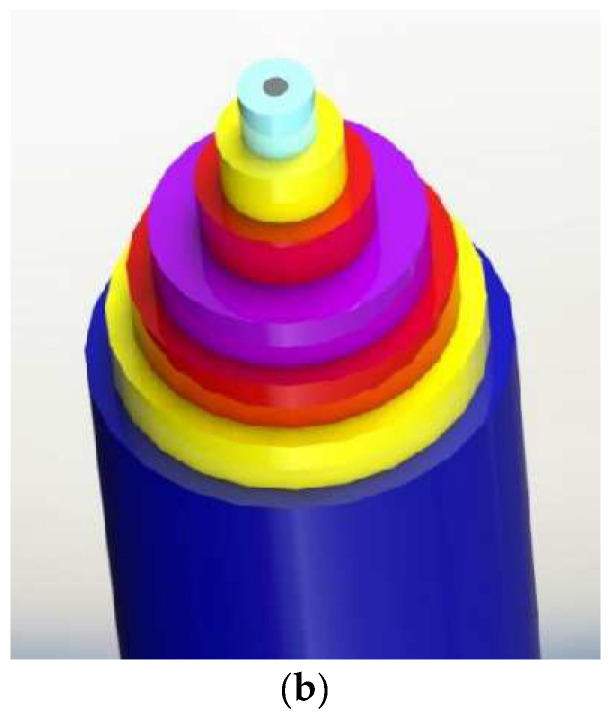
Fabrication of the CSDG MOSFET in a controlled environment [45,46]: (**a**) step-by-step proposed procedure; (**b**) final CSDG MOSFET after fabrication showing concentric cylindrical structure.

The fabrication methodology is the extension of the authors’ work carried out in ref. [6]. The fabrication of the CSDG MOSFET (as shown in Figure 3) was a challenging method since it involved careful involvement of the parameters to create a layer-by-layer approach [16,31]. The core was grown over the concentric discs (as in Figure 3a) (Step I) placed in the chamber base. The core ranges from 2 nm in diameter and with uniform distribution (Step II). The next layer is the gate-1 terminal, which is 6 nm in diameter (Step III). The high-ƙ dielectric layer was placed next to the gate-1 terminal from 6 nm to 10 nm thickness to avoid SCEs (Step IV). The spacer extends for another 4 nm from the dielectric material (Step V). The concentric cylindrical bulk is the largest region of the device which extends from 14 nm to 22 nm in thickness (Step VI). The second spacer layer has an extension from 22 nm to 26 nm with 4 nm in thickness (Step VII). The second high-ƙ dielectric terminal was placed next to the second spacer layer, and it extends to a diameter of 30 nm (Step VIII). The last layer is the gate-2 material, and it acts as a surrounding layer with the largest diameter of 34 nm from the dielectric material (Step IX).

The fabricated CSDG MOSFET can be cut into the desired length, which maintains the L/W ratio and can be scaled and applied to different systems [32,33,34,35]. The desired length of the device is fixed as 100 nm in this work. The transport model used in the fabrication is uncoupled, and phonon scattering is allowed. The results were recorded and compared with the conventional methods of fabrication. The symmetric structure of the CSDG MOSFET was used in simulation to obtain the expected results as described in the next section.

## 4. Results and Discussion

**Figure 4:**
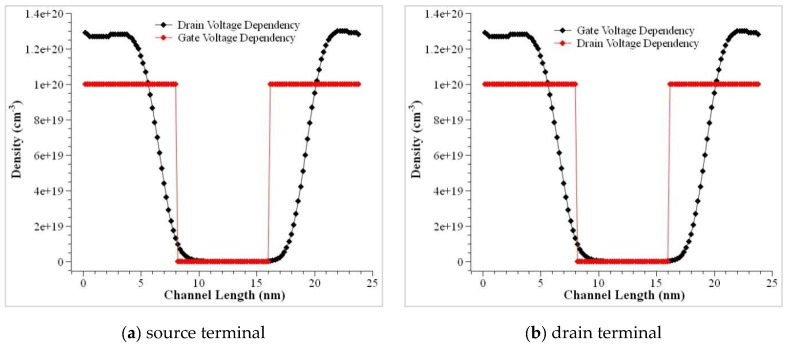
Electron density profile V_D_ versus V_G_ in the CSDG MOSFET. (**a**) source terminal, (**b**) drain terminal.

The mobility of the device was modeled using the doping dependencies, velocity saturation at the transverse electric field, and high-electric field saturation. The I_D_ versus V_G_ curves is plotted below to help understand the characteristics of the CSDG MOSFET device. The double gate structure of the CSDG MOSFET reduces the effect of the SCEs to a greater extent. The SCEs were tackled by the CSDG MOSFET by lowering the subthreshold leakage current. The results were obtained for the symmetric CSDG MOSFET in the three-dimensional space. The results attained from the proposed model were compared with the simulation results, and it is shown in the plots. The bulk of the device is assumed as a floating layer in all the simulations. When the thickness of the oxide layer becomes thin (<10 nm), ion volume inversion takes place due to the quantization of the electrons in the channel. The thickness of the oxide layer is always controlled. If it goes beyond 10 nm, the channel splits, and it results in a larger drain current. The model for the inversion region as in the earlier sections, was calculated by assuming the charge in the channel is fixed. The validity of the model was validated using the simulation of the practical device after fabrication. The electron density profile is shown in Figure 4, and it is evident that it shows the dependency between gate voltage and drain voltage. The minor difference present in the proposed and simulated data is mainly due to the expression in (19a) and the variable nature. This shows that it is suitable for the symmetric doped channel CSDG MOSFET. 

**Figure 5:**
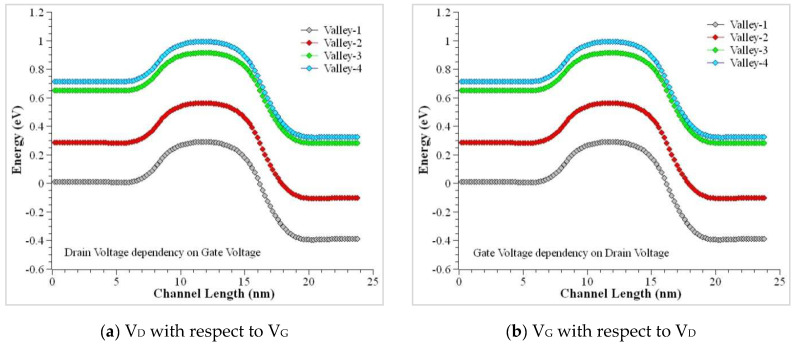
Conduction sub-band profile V_D_ versus V_G_ in the CSDG MOSFET.

**Figure 6:**
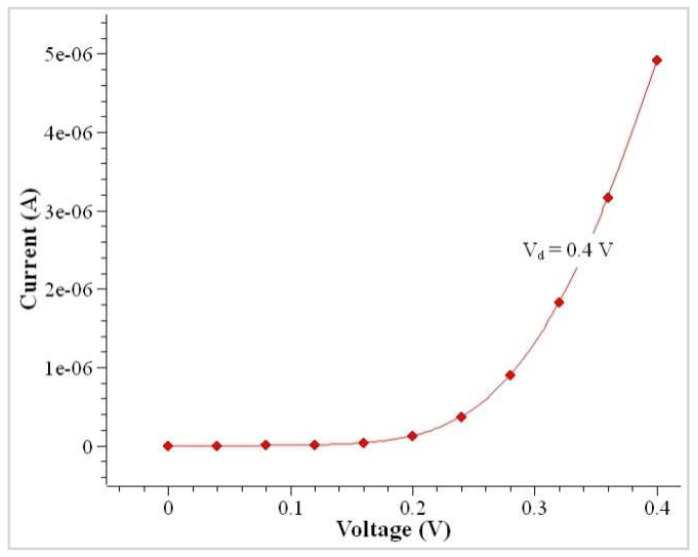
Characteristics of I_D_ versus V_G_ in the CSDG MOSFET.

**Figure 7:**
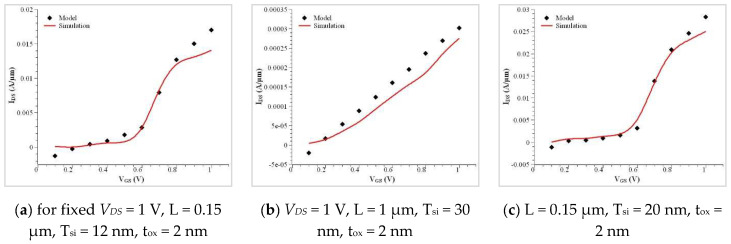
Comparison of doped channel of symmetrical FET between measurements and simulation outputs; I_DS_ versus V_GS_ in the CSDG MOSFET.

For a doped bulk symmetric CSDG MOSFET, variation between the model and simulation value includes the fixed charge value in weak and inversion regions. This uses the drift–diffusion transport model in the CSDG MOSFET of 100 nm channel length. The conduction band profile is illustrated in Figure 5. The characteristics of the cylindrical structure transistor were simulated, and they are plotted in Figure 6. The minor difference present in the drain current measurement is due to the difference in finite mesh density and linear voltage drop. This effect was neglected in the proposed model, making it unsuitable for DG MOSFET. The proposed model is highly suitable for the CSDG MOSFET regime. Figure 7 shows the comparison of the doped channel of symmetrical FET between measurements and simulation outputs (for Tsi = 30 nm); I_DS_ versus V_GS_ in the CSDG MOSFET: (a) For fixed *V_DS_* = 1 V, L = 0.15 μm, T_si_ = 12 nm, t_ox_ = 2 nm; (b) *V_DS_* = 1 V, L = 1 μm, T_si_ = 30 nm, t_ox_ = 2 nm; and (c) L = 0.15 μm, T_si_ = 20 nm, t_ox_ = 2 nm.

**Figure 8:**
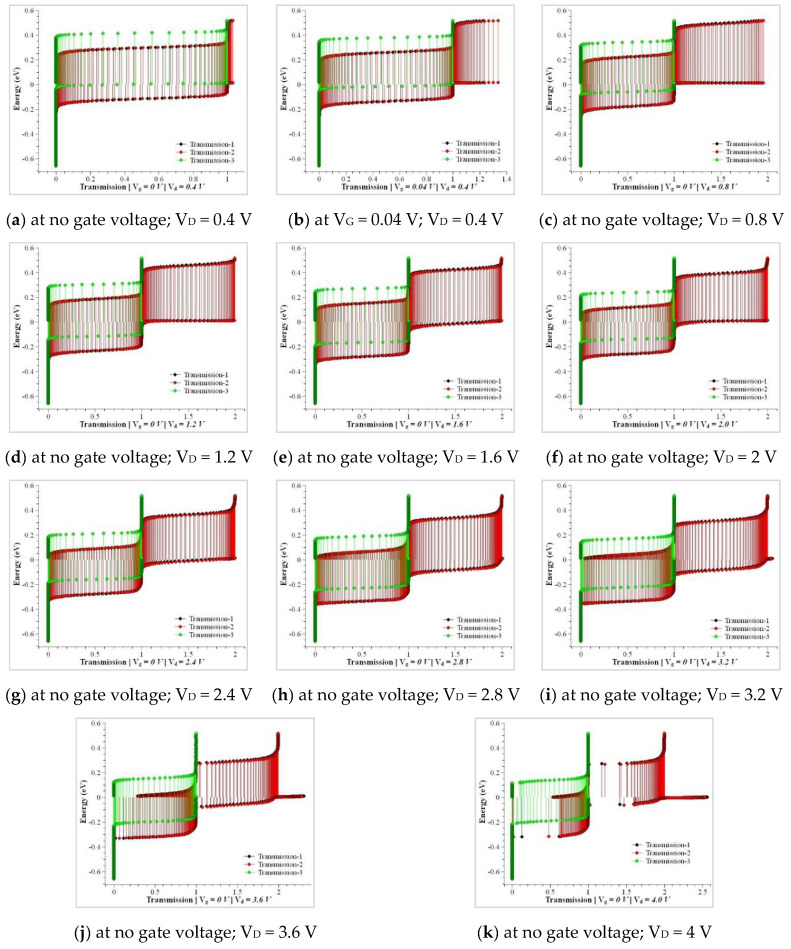
Transmission versus energy based on electron mobility.

**Figure 9:**
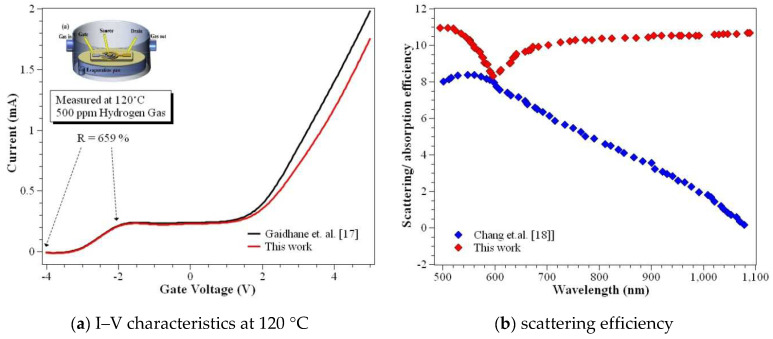
Comparison of technologies based on the proposed CSDG MOSFET model.

**Figure 10:**
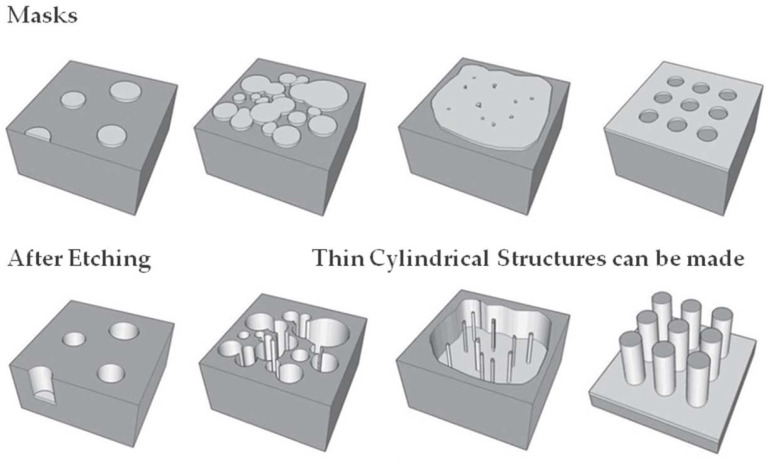
Masks used for cylindrical structure fabrication.

The oxidant concentration plays a major role in performing uniform oxidation in the cylindrical walls of the heterostructure. The maximum oxidant concentration is observed to be 4.37 × 10^16^ cm^−3^ of thickness 0.82 nm for (100) and 2.56 × 10^19^ cm^−3^ of thickness 0.33 nm for (100) at dry oxidation and wet oxidation, respectively. The maximum oxidant concentration of the oxide layer is observed to be 3.90 × 10^16^ cm^−3^ of thickness 0.96 nm for (111) and 2.11 × 10^19^ cm^−3^ of thickness 0.49 nm for (111) at dry oxidation and wet oxidation, respectively. The transmission deviations in the simulation compared to the modeled CSDG MOSFET are plotted in Figure 8a–j with varying drain voltage. Being an extensive analytical approach, the drain current serves the purpose of electron concentration explicitly inside the concentric cylindrical structures. The characteristics of the proposed CSDG MOSFET simulated version were compared with the existing research, and it is illustrated in Figure 9. The fabrication masks for various heterostructures are illustrated in Figure 10. The fabrication masks vary with the unique structures involved in the design of the CSDG MOSFET.

The performance of the device was analyzed for various threshold conditions of the gate voltage and other parameters. The characteristics of the proposed model were compared and presented.

## 5. Conclusions and Future Considerations

Three-dimensional surface potential was presented elaborately considering the mobility of the ions. It was derived from the undoped and the doped channel CSDG MOSFETs. These equations are expedient with Pao-Sah’s integral to solve the drain current across the device length. The proposed model was consistent in terms of three-dimensional surface potential, the mobility for varying electric fields at the gate terminal, and the channel’s thickness. The equations for the surface potential, mobility of ions, and drain current were developed using the physics and mathematical equation simplification noting proper boundary conditions. Better accuracy was achieved using the fitting parameters with boundary conditions.

This work can be further continued by adding a quantum mechanical effect in the Fermi level using several high-κ dielectric materials. In addition, the CSDG MOSFET can be modeled using the effect of electron distribution along the channel and can be validated using various other semiconductor alloys for enhanced performance. The insertion of high-ƙ dielectric in the same structure can give numerous insights towards nano-technological advancements. Going forward, it will be fabricated as a cylindrical structure and tested for various environmental conditions.

## Appendix A

**Table A1:** List of parameters used in modeling.

*L*, *L_eff_*	Channel Length, Effective Channel Length
t_ox_, t_si_	The thickness of the gate oxide, Thickness of Silicon Film
η_i_	Carrier Concentration (Intrinsic Semiconductor)
ε_si_, ε_ox_	The permittivity of Silicon, Permittivity of oxide
φ(x,y,z)	Channel Potential
ϕfb	Fermi Potential of lightly p-doped body
ψ, ψ_s_, ψ_0_	Band Bending Potential
K, T	Boltzmann Constant, Temperature
q	Charge of an electron
*V_t_*, *V_T_*, *V*	The voltage at kT/q, threshold voltage, Fermi level potential
*V_DS_*, *V_GS_*, V_FB_	Drain to source voltage, the gate to source voltage, flat band voltage
N_a_	Acceptor Doping Concentration
H	Electric Field
C_0_	Oxide Capacitance
Q_I_	Inversion Charge Density
μ_eff_,μ_e,si_	Effective Mobility, Mobility of electron in Silicon
E_vertical_, E_horizontal_	Vertical and horizontal Electric Field
ψ_S0_, ψ_SL_, ψ_00_, ψ_0L_	Surface Potential at source and drain, the center of the film

## References

[1] International Roadmap for Devices and Systems (IRDS™) 2021 Edition. https://irds.ieee.org.

[2] Dubey S., Kumar P., Tiwari S. (2010). A two-dimensional model for the potential distribution and threshold voltage of short-channel double-gate metal-oxide-semiconductor field-effect transistors with a vertical Gaussian-like doping profile. J. Appl. Phys..

[3] Zhang X., Jiang Z., Hu J. A novel tri-input Schottky barrier FET exhibiting three-input series switching function. Proceedings of the IEEE 14th International Conference on ASIC (ASICON).

[4] Meriga C., Ponnuri R.T., Krishna B.V., Saidulu S.A., Prakesh M.D. Dual gate junctionless gate-all-around (JL-GAA) FETs using hybrid structured channels. Proceedings of the International Conference for Emerging Technology (INCET).

[5] Gupta S., Pandey N., Gupta R.S. Investigation of Dual-Material Double Gate Junction Less Accumulation-Mode Cylindrical Gate All Around (DMDG-JLAM-CGAA) MOSFET with high-k gate stack for low power digital applications. Proceedings of the IEEE 17th India Council International Conference (INDICON).

[6] Gowthaman N., Srivastava V.M. (2021). Capacitive modeling of cylindrical surrounding double-gate MOSFETs for hybrid RF applications. IEEE Access.

[7] Dargar A., Srivastava V.M. Capacitive model of CSDG MOSFET at pinch-off for switching characteristics. Proceedings of the 10th International Conference on Computing, Communication and Networking Technologies (ICCCNT).

[8] Gowthaman N., Srivastava V.M. Arbitrary alloy semiconductor material based DG MOSFET for high-frequency industrial and hybrid consumer applications. Proceedings of the IEEE AFRICON.

[9] Liu S., Lu L., Ye R., Wu H., Chen H., Wu W., Sun W., Ma S., Liu Y., He B. (2020). Hot-carrier-induced degradation and optimization for 700-V high-voltage lateral DMOS by the AC stress. IEEE Trans. Electron Devices.

[10] Paramasivam P., Gowthaman N., Srivastava V.M. (2021). Design and analysis of InP/InAs/AlGaAs based Cylindrical Surrounding Double-Gate (CSDG) MOSFETs with La_2_O_3_ for 5-nm technology. IEEE Access.

[11] Mukhopadhyay S., Ray P., Deyasi A. Computing gate asymmetric effect on drain current of DG-MOSFET following Ortiz-Conde model. Proceedings of the National Conference on Emerging Trends on Sustainable Technology and Engineering Applications (NCETSTEA).

[12] Deyasi A., Chowdhury A.R., Roy K., Sarkar A. Effect of high-k dielectric on drain current of ID-DG MOSFET using Ortiz-Conde model. Proceedings of the IEEE Electron Devices Kolkata Conference (EDKCON).

[13] Knoll J.S., Son G., Dimarino C., Li Q., Stahr H., Morianz M. (2022). A PCB-embedded 1.2 kV SiC MOSFET half-bridge package for a 22 kW AC-DC Converter. IEEE Trans. Power Electron..

[14] Horii K., Morikawa R., Katada R., Hata K., Sakurai T., Hayashi S.-I., Wada K., Omura I., Takamiya M. Equalization of DC and surge components of drain current of two parallel-connected SiC MOSFETs using single-input dual-output digital gate driver IC. Proceedings of the IEEE Applied Power Electronics Conference and Exposition (APEC).

[15] Gowthaman N., Srivastava V.M. (2021). Parametric analysis of CSDG MOSFET with La_2_O_3_ gate oxide: Based on electrical field estimation. IEEE Access.

[16] Robertson J. (2004). High dielectric constant oxides. Eur. Phys. J. Appl. Phys..

[17] Gaidhane A.D., Pahwa G., Verma A., Chauhan Y.S. (2020). Gate-induced drain leakage in negative capacitance FinFETs. IEEE Trans. Electron Devices.

[18] Chang P.C., Hsiao C.J., Lumbantoruan F.J., Wu C.H., Lin Y.K., Lin Y.C., Sze S.M., Chang E.Y. (2018). InGaAs junctionless FinFETs with self-aligned Ni-InGaAs S/D. IEEE J. Electron Devices Soc..

[19] Sze S.M., Li Y., Ng K.K. (2021). Physics of Semiconductor Devices.

[20] Gowthaman N., Srivastava V.M. InP/AlGaAs based CSDG MOSFET with Au/Pt Gate materials for high frequency/hybrid applications. Proceedings of the XXX International Scientific Conference Electronics (ET).

[21] García I., Rey-Stolle I., Galiana B., Algora C. (2007). Analysis of tellurium as the n-type dopant in GaInP: Doping, diffusion, memory effect, and surfactant properties. J. Cryst. Growth.

[22] Ansari M.H.R., Cho S., Lee J.H., Park B.G. (2021). Core-shell dual-gate nanowire memory as a synaptic device for neuromorphic application. IEEE J. Electron Devices Soc..

[23] Passlack M. (2005). Development methodology for high-κ gate dielectrics on III–V semiconductors: GdxGa0.4-xO0.6/Ga2O3 dielectric stacks on GaAs. J. Vac. Sci. Technol. B Microelectron. Nanometer Struct. Process. Meas. Phenom..

[24] Varadharajan S., Kaya S. Study of dual-gate SOI MOSFETs as RF mixers. Proceedings of the International Semiconductor Device Research Symposium (ISDRS).

[25] Kumar A., Srinivas P.S.T.N., Tiwari P.K. Compact drain current model of silicon-nanotube-based double gate-all-around (DGAA) MOSFETs incorporating short channel effects. Proceedings of the 14th Nanotechnology Materials and Devices Conference (NMDC).

[26] Srivastava V.M., Yadav K.S., Singh G. (2010). Application of VEE Pro software for measurement of MOS device parameters using C-V curve. Int. J. Comput. Appl..

[27] Mao C., Solis D.J., Reiss B.D., Kottmann S.T., Sweeney R.Y., Hayhurst A., Georgiou G., Iverson B., Belcher A.M. (2004). Virus-based toolkit for the directed synthesis of magnetic and semiconducting nanowires. Science.

[28] Lin J., Antoniadis D.A., Del Alamo J.A. (2014). Off-state leakage induced by band-to-band tunneling and floating-body bipolar effect in InGaAs quantum-well MOSFETs. IEEE Electron Device Lett..

[29] Lin J., Antoniadis D.A., Del Alamo J.A. (2015). Physics and mitigation of excess OFF-state current in InGaAs quantum-well MOSFETs. IEEE Trans. Electron Devices.

[30] Lim S.K., Crawford S., Haberfehlner G., Gradecak S. (2013). Controlled modulation of diameter and composition along individual III–V nitride nanowires. Nano Lett..

[31] Kim S., Kim S.K., Shin S., Han J.H., Geum D.M., Shim J.P., Lee S., Kim H., Ju G., Song J.D. (2019). Highly stable self-aligned Ni-InGaAs and non-self-aligned Mo contact for monolithic 3-D integration of InGaAs MOSFETs. IEEE J. Electron Devices Soc..

[32] Mo J., Lind E., Wernersson L.E. (2014). Asymmetric InGaAs/InP MOSFETs with source/drain engineering. IEEE Electron Device Lett..

[33] Zhang X., Guo H., Lin H.Y., Ivana, Gong X., Zhou Q., Lin Y.R., Ko C.H., Wann C.H., Yeo Y.C. (2011). Reduction of off-state leakage current in In0.7Ga0.3As channel n-MOSFETs with self-aligned Ni-InGaAs contact metallization. Electrochem. Solid-State Lett..

[34] Goto S., Matsunaga T., Chen J.J., Makishi W., Esashi M., Haga Y. Fabrication techniques for multilayer metalization and patterning, and surface mounting of components on cylindrical substrates for tube-shaped micro-tools. Proceedings of the International Conference on Microtechnologies in Medicine and Biology.

[35] Sallese J.M., Jazaeri F., Barbut L., Chevillon N., Lallement C. (2013). A common core model for junctionless nanowires and symmetric double-gate FETs. IEEE Trans. Electron Devices.

[36] Holtij T., Graef M., Hain F.M., Kloes A., Iñíguez B. (2014). Compact model for short-channel junctionless accumulation mode double-gate MOSFETs. IEEE Trans. Electron Devices.

[37] Duarte J.P., Choi S.J., Moon D.I., Choi Y.K. (2012). A Non-piece-wise model for long-channel junctionless cylindrical nanowire FETs. IEEE Electron Device Lett..

[38] Villa J., Ramiro I., Ripalda J.M., Tobías I., García-Linares P., Antolin E., Martí A. (2021). Contribution to the study of sub-bandgap photon absorption in quantum dot InAs/AlGaAs intermediate band solar cells. IEEE J. Photovolt..

[39] Shahrjerdi D., Rotter T., Balakrishnan G., Huffaker D., Tutuc E., Banerjee S.K. (2008). Fabrication of self-aligned enhancement-mode In0.53Ga0.47As MOSFETs with TaN/HfO2/AlN gate stack. IEEE Electron Device Lett..

[40] Taur Y., Ning T.H. (2021). Fundamentals of Modern VLSI Devices.

[41] Dorow C., O’Brien K., Naylor C.H., Lee S., Penumatcha A., Hsiao A., Tronic T., Christenson M., Maxey K., Zhu H. (2021). Advancing Monolayer 2D nMOS and pMOS transistor integration from growth to van der Waals interface engineering for ultimate CMOS scaling. IEEE Trans. Electron Devices.

[42] Fu H., Fu K., Chowdhury S., Palacios T., Zhao Y. (2021). Vertical GaN power devices: Device principles and fabrication technologies—Part II. IEEE Trans. Electron Devices.

[43] Kim S., Kim M., Ryu D., Lee K., Kim S., Lee J., Lee R., Kim S., Lee J.H., Park B.G. (2020). Investigation of electrical characteristic behavior induced by the channel-release process in stacked nanosheet gate-all-around MOSFETs. IEEE Trans. Electron Devices.

[44] Wang Y., Wang W., Abbasi H.N., Chang X., Zhang X., Zhu T., Liu Z., Song W., Chen G., Wang H. (2020). LiF/Al₂O₃ as Dielectrics for MOSFET on a single-crystal hydrogen-terminated diamond. IEEE Electron Device Lett..

[45] Srivastava V.M., Yadav K.S., Singh G. (2012). Drain current and noise model of cylindrical surrounding double-gate MOSFET for RF switch. Procedia Eng..

[46] Gowthaman N., Srivastava V.M. (2022). Design of Concentric Cylindrical Surrounding Double-Gate (CSDG) MOSFETs—A Fabrication Perspective in Nanoscale Regime.

